# Prevalence and factors associated with covid-19 vaccine hesitancy in Maranhão, Brazil

**DOI:** 10.11606/s1518-8787.2021055003417

**Published:** 2021-04-15

**Authors:** Bruno Luciano Carneiro Alves de Oliveira, Marcos Adriano Garcia Campos, Rejane Christine de Sousa Queiroz, Maria Teresa Seabra Soares de Britto e Alves, Bruno Feres de Souza, Alcione Miranda dos Santos, Antônio Augusto Moura da Silva

**Affiliations:** I Universidade Federal do Maranhão Departamento de Medicina I São LuísMA Brasil Universidade Federal do Maranhão. Departamento de Medicina I. São Luís, MA, Brasil; II Secretaria Municipal de Saúde São LuísMA Brasil Secretaria Municipal de Saúde. São Luís, MA, Brasil; III Universidade Federal do Maranhão Departamento de Saúde Pública São LuísMA Brasil Universidade Federal do Maranhão. Departamento de Saúde Pública. São Luís, MA, Brasil; IV Universidade Federal do Maranhão Departamento de Engenharia da Computação São LuísMA Brasil Universidade Federal do Maranhão. Departamento de Engenharia da Computação. São Luís, MA, Brasil

**Keywords:** Coronavirus Infections, prevention & control, Vaccination Refusal, psychology, Health Knowledge, Attitudes, Practice, Health Surveys

## Abstract

**OBJECTIVES:**

To estimate the prevalence and factors associated with hesitancy in getting the vaccine against SARS-CoV-2 in Maranhão, Brazil.

**METHODS:**

This is a cross-sectional population-based study conducted from October 19 to 30, 2020. The estimates were calculated based on clustering, stratification, and non-response. A three-stage sampling was adopted, considering stratum, census tracts, and domicile. After systematic analysis, thirty sectors were selected in each stratum, totaling 150 sectors. Each sector contained a fixed number of 34 households, thus totaling 5,100 households. One individual within each household (resident for at least six months and aged one year or more) was selected by a simple random sampling. We questioned participants about their vaccination intention. Univariate association between independent variables and the outcome were verified using descriptive analysis (weighted frequencies) and Pearson's chi-square test (p < 0.05). Robust multivariate analysis was performed using a three-level hierarchical model.

**RESULTS:**

We found 17.5% (95%CI 16.1–19.1%) of the 4,630 individuals interviewed to report hesitancy to be vaccinated against covid-19. After final model adjustment, vaccination hesitancy was statistically higher among residents of the cities of Imperatriz (24.0%; RP = 1.48; IC95% 1.09–2.02) and municipalities of the Grande Ilha de São Luís (20.7%; RP = 1.34; 95%CI 1.02–1.76), female individuals (19.8%; RP = 1.44; 95%CI 1.20–1.75), older adults (22.8%; RP = 1.79; IC95% 1.30–2.46), evangelicals (24.1%; RP = 1.49; 95%CI 1.24–1.79), and those without reported symptoms (18.6%; RP = 1.24; 95%CI 1.02–1.51). We found no statistical differences for other socioeconomic and demographic characteristics, as well as variables related to the labor market, behaviors, and health conditions of the interviewees.

**CONCLUSION:**

The prevalence of vaccine hesitancy in Maranhão and its association with individual, contextual, and clinical factors enable us to identify the groups and contexts of greatest resistance, requiring special attention from public strategies to ensure wide vaccination.

## INTRODUCTION

In the course of the pandemic, state and local governments in Brazil mobilized strategic actions to control the exposure to SARS-CoV-2 virus. To diminish the magnitude of the disease, the country implemented an extensive list of non-pharmacological interventions (NPI)^1^. However, a permanent solution is more likely to be provided by developing, making available, and implementing effective, safe, and high-quality vaccines against the virus^2^.

Vaccination is one of the most cost-effective ways to prevent diseases. Currently, immunization is estimated to prevent 2–3 million deaths every year and could possibly avoid an additional 1.5 million if global vaccination coverage was improved^3^. Brazil has one of the world's largest public vaccination programs, offering a wide range of vaccines and other special immunobiologicals for specific audiences through regular routine schedule and campaigns. Yet, the program distinctive high coverage has fallen over the past years. In 2019, 89,776,476 million doses of vaccines were administered. Coverage rates ranged from 61.2% in Rio de Janeiro and 63.2% in Maranhão to 90.0% in Mato Grosso do Sul. In 2020, the pandemic scenario led to vaccination suspension, reducing the number of doses (n = 57,519,127) and vaccination rates (32.2% in Amapá, 39.2% in Maranhão, and 66.1% in the Federal District)^4^, causing new outbreaks of vaccine-preventable diseases^5^.

Although the epidemiological and socioeconomic benefits of immunization programs are well-known, the debate over vaccine hesitancy (reluctance, indecision, or refusal to vaccinate despite availability of vaccination services) has grown worldwide. Such hesitancy poses a global threat to the progress made in combating vaccine-preventable diseases, thus becoming a central issue for immunization programs^6^^-^^8^. Vaccine hesitancy owes to complex causes of varying forms and intensities, based on when and where vaccination occurs, which vaccine is involved, and on which public it is administered^9^^,^^10^. In 2019, the World Health Organization (WHO) included vaccine hesitancy as one of the ten leading threats to global health, proposing strategies for its mitigation along with other non-governmental institutions^3^^,^^6^.

Worldwide, several candidate vaccines against the novel coronavirus are in various stages of development. Some, in more advanced stages of testing, will be feasible for administration between the end of 2020 and the first months of 2021 in most upper and middle income countries^11^^,^^12^. Despite the advance, political polarizations, conspiracy theories, anti-vaccine movements, and vaccine-related concerns have rapidly increased in social networks and traditional media during the covid-19 pandemic. Unverified/incomplete information, rumors, and memes about coronavirus vaccines and origins reach many people faster than complex scientific information^11^^,^^13^^,^^14^, possibly affecting vaccine confidence and acceptance^9^^,^^11^. New vaccines may have restored the hopes of returning to pre-covid normality, but they also raise questions about unknown effects and sparked speculation about potential mandatory vaccination^11^^,^^13^^,^^14^.

Thus, local authorities must employ the available resources to deal with complex ethical and health issues for overcoming vaccine hesitancy. For that, they must know which groups are more resistant to immunization and which strategies should be implemented for preparing the population for vaccination, ensuring high coverage rates and homogeneity levels among different groups and localities. Knowing the prevalence and characteristics associated with vaccine hesitancy in the Brazilian context may help ensuring the effectiveness of immunization programs and campaigns against covid-19.

This study estimated the prevalence and factors associated with vaccine hesitancy in the context of the covid-19 pandemic using a questionnaire on SARS-CoV-2 virus infection, conducted in Maranhão, Brazil.

## METHODS

### Study Type and Population

This is a cross-sectional study conducted with data from the population-based household serological survey entitled “Prevalence of SARS-CoV-2 infection in Maranhão, Brazil,” developed in cooperation with the Universidade Federal do Maranhão and the Health Department of Maranhão, from October 19 to 30, 2020. The municipalities of Maranhão were divided into five strata according to 2019 municipal population reported by the Brazilian Institute of Geography and Statistics (IBGE)^15^: Grande Ilha; less than 20,000 inhabitants; from 20,000 to 100,000 inhabitants; more than 100,000 inhabitants, and Imperatriz (second largest in population and economy). The Grande Ilha comprised the state capital, São Luís, and three other neighboring cities.

### Sample

Sample size was calculated based on the estimated prevalence of SARS-Cov-2 infection of the first survey conducted in the state^16^. In each stratum, sample size was estimated from the following equation:

$$n=\frac{N}{N-1}*P*Q*\frac{1}{CV^{2*}P^{2*}\frac{P^{*}Q}{N-1}},$$

Where *N* is the population in each stratum, P the prevalence, and *CV* the estimates coefficient of variation of the expected prevalence within the strata. A design effect was considered at 2. The study sample consisted of 5,001 individuals: 872 in Stratum 1 (four municipalities); 1,236 in Stratum 2 (122 municipalities); 612 in Stratum 3 (85 municipalities); 1,022 in Stratum 4 (five municipalities); and 1,021 in Stratum 5 (one municipality).

Sample selection was performed in three stages. In the first, census sectors were selected within each stratum. Within sectors, households were selected. Finally, only one individual residing within each household was selected.

In each stratum, sectors were selected from a systematic random sampling, proportional to the number of permanent private households. Thirty sectors were selected, totaling 150 sectors. Sectors with less than 200 households in the 2010 census were grouped with others, respecting their continuity, so that each clustered sector had at least 200 households. The number of sectors and households was retrieved from the 2010 demographic census^17^.

Thirty-four households within each sectors or clusters were selected by systematic sampling, totaling 5,100 households. Likewise, one individual within each household was selected from a simple random sampling based on a list of eligible residents compiled during the interview (resident for at least six months and aged one year or more), totaling 5,100 individuals. The final sample reached a 65.4% response rate (n = 4,630), and the final sample weight considered the three-stage sampling and the response rate.

### Data Collection

Data were collected by means of a questionnaire and by drawing 5mL of blood from the chosen individual, and then recorded in the EpiCollect platform from a mobile device^18^.

A laboratory technician and an interviewer, both using personal protective equipment (PPE), comprised data collection teams. Blood was collected to detect the presence of total antibodies (IgM, IgG, and IgA) against SARS-CoV-2 by serological testing using the electro-chemiluminescence immunoassay (ECLIA), the cobas e 601^®^ immunoassay analyzer module (Roche Diagnostics), and Elecsys^®^ Anti-SARS-CoV-2 (Roche Diagnostics)^19^^,^^20^.

### Study variables

In our study, covid-19 vaccine hesitancy was the outcome variable, assessed by the question: “Would you take the covid-19 vaccine if it were available to the population?”. Possible answers were: “yes,” “no,” and “I don't know”. For children, the guardian's decision was considered. For reflecting refusal or indecision about immunization, “no” and “I don't know” were classified as “vaccine hesitancy.”

A set of independent variables was evaluated. Socioeconomic and demographic variables were: gender, age group (1–19, 20–59, ≥ 60), color/ethnicity (white, mixed race, black), education level (up to complete primary education, complete secondary education, complete tertiary education), household income in reais (< 1,000, 1,000 a< 2,000, ≥ 2,000), health insurance (yes, no), religion (catholic, evangelical, no religion, others), residents per household (1, 2, ≥ 3), and use of public transportation during the pandemic (no, yes). Variables related to the labor market after the pandemic onset were: keep working face-to-face (yes, no, didn't work outside the home before the pandemics), works remotely, even if partially (yes, no, didn't work), lost their job or experienced a reduction in household income (yes, no, didn't work), received *Bolsa Família* aid (yes, no), received emergency aid (yes, no), and received unemployment insurance benefits (yes, no, didn't work). As for variables related to individuals’ behaviors and health conditions, they comprised compliance with non-pharmacological interventions from the pandemic onset (March 2020 was used as reference) until the survey date (October 2020), that is: social isolation (yes, no), use of masks (yes, no), hand hygiene (yes, no), and physical distancing (yes, no); frequency of symptoms^6^ possibly related to covid-19 (no symptoms, 1 to 2, ≥ 3); having performed covid-19 diagnostic testing (RT-PCR, rapid test, or serology) before the investigation (yes, no); and SARS-CoV-2 serology identified in the survey (positive, negative).

### Statistical Analysis

Statistical analysis was performed in the Stata^®^ 14 software, considering the complex sampling design and sample weighting. The prevalence of vaccination hesitancy and respective 95% confidence intervals (95%CI) were estimated based on independent variables, whose univariate association was assessed using the Pearson's chi-square test at 5% significance level. Multivariate Poisson analysis was performed using hierarchical modeling (Figure). Explanatory variables were arranged at three levels: distal (socioeconomic and demographic characteristics), intermediate (work-related characteristics after the pandemic onset), and proximal (behaviors and health conditions). Variables were analyzed in blocks. The final model was acquired by keeping variables with p < 0.05 in the analyses while adding those from the next block. Prevalence ratio (PR) and 95%CI were estimated using Poisson regression with robust variance (α = 5%). Estimates for variables within each block are presented.

**Figure:**
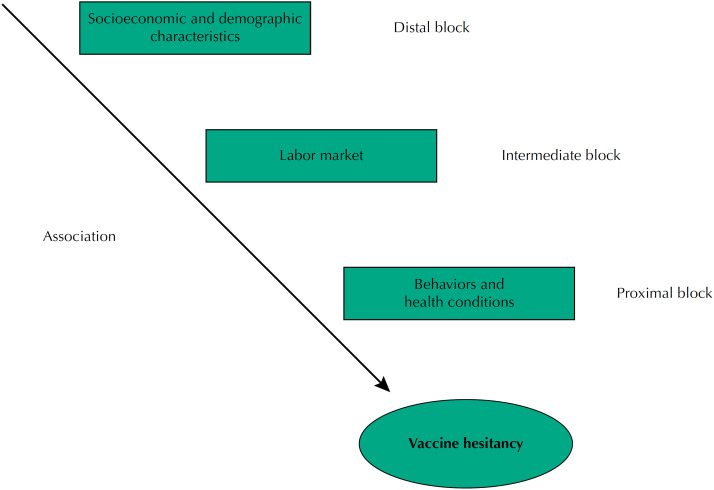
Hierarchical model for analyzing factors associated with covid-19 vaccine hesitancy, if it were already available, according to a survey on SARS-CoV-2 virus infection conducted in Maranhão, Brazil, 2020.

### Ethical Aspects

This survey was approved by the Research Ethics Committee of the Hospital Carlos Macieira of the Department of Health of Maranhão, under no. CAAE 34708620.2.0000.8907.

## RESULTS

The prevalence of covid-19 vaccine hesitancy was 17.5% (95%CI 16.1–19.1). When compared to cities with less than 20,000 inhabitants (14.0%; 95%CI 11.7–16.6), this prevalence was statistically higher (p = 0.0036) in Imperatriz (24.0%; 95%CI 18.1–31.1) and in the municipalities of Grande Ilha de São Luís (20.7%; 95%CI 17.6–24.3). Hesitancy was also higher among women (19.8%; 95%CI 17.9–21.9; p = 0.0001), older adults (22.8%; 95%CI 19.5–26.4; p = 0.0003), and evangelicals (24.1%; 95%CI 20.7–27.9; p = 0.0001). For the other socioeconomic and demographic characteristics, the prevalence of vaccine hesitancy approached the state estimate, but without statistical association (p > 0.05), as shown in Table 1.

**Table 1:** Prevalence of covid-19 vaccine hesitancy, if it were available to the population, according to socioeconomic and demographic characteristics of the interviewees in the serological survey conducted in Maranhão, Brazil, 2020.

Variables		Vaccine hesitancy				p^a^
		Yes		No		
		%	95%CI	%	95%CI	
General population		17.5	16.1–19.1	82.5	80.9–83.9	
Cities stratum						
	< 20,000 inhabitants	14.0	11.7–16.6	86.0	83.4–88.3	0.0036
	20 to 100,000 inhabitants	16.6	14.1–19.5	83.4	80.5–85.9	
	> 100,000 inhabitants	16.9	14.4–19.7	83.1	80.3–85.6	
	Imperatriz	24.0	18.1–31.1	76.0	68.9–81.9	
	Grande Ilha^b^	20.7	17.6–24.3	79.3	75.7–82.4	
Gender						
	Female	19.8	17.9–21.9	80.2	78.1–82.2	0.0001
	Male	13.2	11.3–15.5	86.8	84.5–88.7	
Age group (in years)						
	0–19	13.1	10.0–16.9	87.9	83.1–90.0	0.0003
	20–59	16.7	14.9–18.7	83.3	81.3–85.1	
	≥ 60	22.8	19.5–26.4	77.2	73.6–80.5	
Color/ethnicity^c^						
	White	17.4	14.2–21.2	82.6	78.8–85.8	0.166
	Brown/Mixed race	18.0	16.1–20.0	82.0	80.0–83.9	
	Black	14.8	11.8–18.5	85.2	81.5–88.2	
Education level^d^						
	Complete primary education	17.8	16.0–19.8	82.2	80.2–84.0	0.231
	Complete secondary education	18.2	15.5–21.4	81.8	78.6–84.6	
	Complete tertiary education	13.7	10.3–18.0	86.3	82.0–89.7	
Household income (R$)^d^						
	< 1,000	15.7	13.0–19.0	84.3	81.0–87.0	0.3731
	From 1,000 to < 2,000	18.6	16.4–21.0	81.4	79.0–83.6	
	≥ 2,000	17.5	14.9–20.3	82.5	79.7–85.1	
Has health insurance						
	No	17.4	15.9–19.0	82.6	81.0–84.1	0.4879
	Yes	19.5	14.2–26.1	80.5	73.9–85.8	
Religion						
	Catholic	15.2	13.6–17.0	84.8	83.0–86.4	0.0001
	Evangelical	24.1	20.7–27.9	75.9	72.1–79.3	
	Does not have one	20.0	14.2–27.4	80.0	72.6–85.8	
	Others	12.7	5.6–26.3	87.3	73.7–94.4	
Number of residents in the household						
	1	21.0	17.2–25.3	79.0	74.7–82.8	0.120
	2	19.1	16.5–21.9	80.9	78.1–83.5	
	≥ 3	16.8	15.0–18.8	83.2	81.2–85.0	
Used public transportation during the pandemic						
	No	16.4	14.5–18.4	83.6	81.6–85.5	0.074
	Yes	19.2	16.9–21.6	80.8	78.4–83.1	

95%CI: 95% confidence interval.

a Pearson chi-square test.

b Includes the capital, São Luís.

c Does not account for Asian and indigenous ethnicities due to low frequency.

d n differs from 4,563.

We verified no statistically significant association (p *>* 0.05) between vaccine refusal and factors associated with the labor market and interviewees’ socioeconomic vulnerability before the pandemic onset (Table 2). Table 3 shows interviewees’ behaviors and health conditions toward vaccine hesitancy, without statistical association with the variables assessed. For these variables, the prevalence of refusal approached that estimated for the overall state.

**Table 2:** Prevalence of covid-19 vaccine hesitancy, if it were already available to the population, according to characteristics related to the labor market after the pandemic onset reported by interviewees in the serological survey conducted in Maranhão, Brazil, 2020.

Variables		Vaccine hesitancy				p^a^
		Yes		No		
		%	95%CI	%	95%CI	
Keeps working face-to-face						
	No	17.5	15.1–20.2	82.5	79.8–84.9	0.0564
	Yes	13.7	10.8–17.2	86.3	82.8–89.2	
	Didn't work outside the home before the pandemic	18.8	16.7–21.2	81.2	78.8–83.3	
Works remotely, even if partially						
	No	17.1	14.9–19.7	82.9	80.3–85.1	0.4353
	Yes	15.1	11.4–19.7	85.0	80.3–88.7	
	Didn't work before the pandemic	18.2	16.2–20.5	81.8	79.5–83.4	
Lost the job or experienced a reduction in household income						
	No	17.2	15.1–19.6	82.8	80.4–84.9	0.8049
	Yes	16.5	12.5–21.4	83.5	78.7–87.5	
	Didn't work before the pandemic	18.0	15.8–20.4	82.0	79.6–84.2	
Received *Bolsa Família* aid						
	No	17.9	16.1–19.8	82.1	80.2–83.9	0.5468
	Yes	16.9	14.4–19.7	83.1	80.3–85.6	
Received emergency aid						
	No	18.5	16.3–21.0	81.5	79.1–83.7	0.2632
	Yes	16.8	14.9–18.9	83.2	81.1–85.1	
Received unemployment insurance						
	No	16.3	14.5–18.3	83.7	81.7–85.5	0.1791
	Yes	14.9	0.7–30.4	85.1	69.6–93.5	
	Didn't work before the pandemic	19.4	17.0–22.0	80.6	78.0–83.0	

95%CI: 95% confidence interval.

a Pearson chi-square test.

**Table 3:** Prevalence of covid-19 vaccine hesitancy, if it were available to the population, according to the behaviors and health conditions of the interviewees in the serological survey conducted in Maranhão, Brazil, 2020.

Variables		Vaccine hesitancy				p^a^
		Yes		No		
		%	95%CI	%	95%CI	
Compliance with non-pharmacological interventions from the pandemic onset until the survey date						
Social isolation^b^						
	No	18.1	16.3–20.0	81.9	80.0–83.7	0.2968
	Yes	16.3	13.8–19.1	83.7	80.9–86.2	
Use of masks^c^						
	No	18.1	16.2–20.2	81.9	79.8–83.8	0.3828
	Yes	16.7	14.6–19.3	83.3	80.9–85.4	
Hand hygiene^d^						
	No	17.9	16.1–20.0	82.1	80.0–84.0	0.4760
	Yes	16.8	14.6–19.3	83.2	80.7–85.4	
Physical distancing^e^						
	No	17.4	15.7–19.2	82.6	80.8–84.3	0.7608
	Yes	17.9	15.2–21.0	82.1	79.4–84.8	
Received medical diagnosis^f^						
	No	17.4	15.9–19.0	82.6	81.0–84.1	0.5401
	Yes	19.3	13.8–26.4	80.7	73.6–86.2	
Frequency of symptoms possibly related to covid-19^g^						
	No symptoms	18.6	16.6–20.9	81.4	79.1–83.4	0.3990
	One to two symptoms	16.4	12.5–21.2	83.6	78.8–87.5	
	Three or more symptoms	16.6	14.3–19.1	83.4	80.9–85.7	
Has undergone a covid-19 diagnostic testing^h^ before the survey						
	No	17.7	16.1–19.3	82.3	80.7–83.9	0.6455
	Yes	16.5	12.4–21.6	83.5	78.4–87.6	
SARS-CoV-2 serology identified in the survey						
	Positive	18.8	16.4–21.5	81.2	78.5–83.6	0.1894
	Negative	16.7	15.0–18.7	83.3	81.3–85.1	

1 95%CI: 95% confidence interval.

a Pearson's chi-square test.

b Never or almost never leaves home – at most once every 15 days.

c Wears masks every time he/she leaves home and never or hardly ever removes it from the face.

d Performs hand hygiene six times or more per shift.

e Never or hardly ever stays less than 1.5 meters away from other people.

f Suspected covid19.

g The following symptoms were considered: fever, chills, sore throat, cough, dyspnea, anosmia, ageusia, diarrhea, nausea/vomiting, headache, fatigue, and myalgia. They were classified into: no symptoms; one or two symptoms, provided they were not anosmia/hyposmia or ageusia/dysgeusia; three or more symptoms (including anosmia/hyposmia or ageusia/dysgeusia).

h RT-PCR, rapid test, or serology.

After adjusting the hierarchical multivariate model, we found residents of Imperatriz (PR = 1.48; 95%CI 1.09–2.02) and Grande Ilha (PR = 1.34; 95%CI 1.02–1.76), female (PR = 1.44; 95%CI 1.20–1.75), older adults (PR = 1.79; 95%CI 1.30–2.46), evangelicals (PR = 1.49; 95%CI 1.24–1.79), and individuals who experienced no symptoms during the pandemic (PR = 1.24; 95%CI 1.02–1.51) to be more likely to report vaccine hesitancy (Table 4).

**Table 4:** Factors associated with covid-19 vaccine hesitancy, if it were already available to the population, among interviewees in the second phase of the serological survey conducted in Maranhão, Brazil, 2020.

Variables		Distal block		Distal + intermediate block		Distal + intermediate + proximal block	
		RP^a^	95%CI	RP^b^	95%CI	RP^c^	95%CI
Cities stratum							
	< 20,000 inhabitants	1.00	-----				
	20 to 100,000 inhabitants	1.11	0.88–1.40				
	> 100,000 inhabitants	1.15	0.91–1.46				
	Imperatriz	1.48	1.09–2.02				
	Grande Ilha	1.34	1.02–1.76				
Gender							
	Male	1.00	-----				
	Female	1.44	1.20–1.75				
Age group (in years)							
	0–19	1.00	-----				
	20–59	1.29	0.95–1.74				
	≥ 60	1.79	1.30–2.46				
Religion							
	Catholic	1.00	-----				
	Evangelical	1.49	1.24–1.79				
	Does not have one	1.38	0.96–2.00				
	Others	0.85	0.40–1.82				
Frequency of symptoms possibly related to covid-19^d^							
	Three or more symptoms					1.00	-----
	One to two symptoms					1.08	0.80–1.47
	No symptoms					1.24	1.02–1.51

95%CI: confidence interval; PR: prevalence ratio obtained by Poisson's regression.

a Adjusted by each other.

b Adjusted for gender, stratum, age group, ethnicity, religion, number of residents, and use of public transportation. We found no association among variables from intermediate factors.

c Adjusted for gender, stratum, age group, religion, compliance with non-pharmacological interventions, covid-19 testing before and during the survey.

d The following symptoms were considered: fever, chills, sore throat, cough, dyspnea, anosmia, ageusia, diarrhea, nausea/vomiting, headache, fatigue, and myalgia. They were classified into: no symptoms; one or two symptoms, provided they were not anosmia/hyposmia or ageusia/dysgeusia; three or more symptoms (including anosmia/hyposmia or ageusia/dysgeusia).

## DISCUSSION

Our results indicate that, if the covid-19 vaccine was available, most interviewees would like to take it. However, we also verified a significant prevalence of vaccine hesitancy among respondents. In the adjustment model, this prevalence was associated with individual, contextual, and clinical characteristics, such as being female, older adult, evangelical, living within the two strata from the cities with the largest population, and not presenting covid-19-related symptoms during the pandemic.

Immunization decisions comprise a complex behavioral phenomenon, involving cultural, geographical, psychosocial, economic, religious, political, cognitive, and gender factors. The causes of vaccine hesitancy are classified into three interrelated categories: lack of confidence (in their efficacy and safety, in the health system providing them, or in managers and policymakers’ motivations to recommend them), complacency (low risk perception of acquiring immunopreventable diseases, deeming vaccination unnecessary), and lack of convenience (immunization services availability, accessibility, and appeal, including time, place, language, and cultural contexts)^21^^,^^22^.

Research conducted in other countries have also verified a relevant prevalence of hesitancy against SARS-CoV-2 vaccine. A survey conducted with a random sample of 13,426 individuals from 19 countries (whose sum corresponds to 55% of the world's population) found a 28.5% vaccine hesitancy, ranging from 11.4% in China to 45.1% in Russia^9^. In this study, Brazil had the second lowest estimated prevalence (14.7%), below that found in other middle-income countries (India, Mexico and South Africa)^9^ and in Maranhão. The prevalence of vaccine hesitancy was 14.0% in Turkey^23^, 31.0% in the United Kingdom^23^, and ranged from 33.0%^10^ to 42.4%^24^ in the US. As for Israel^25^, studies found a 25.0% prevalence in the overall population, 22.0% in physicians, and 39.0% in nurses.

These results show that vaccine hesitancy varies according to the context. In our research, the capital strata and that of the second largest and richest city in Maranhão presented higher prevalence and likelihood of vaccine refusal than smaller population strata, reaching values close to those observed in countries such as the USA, South Korea, Mexico, India, and Spain^9^. A survey conducted in the US observed a geographic variation in the prevalence of covid-19 vaccine rejection within the country (25.0% to 50.0%)^10^, which may be explained by different epidemic dynamics in each stratum, socioeconomic status, and healthcare access. The covid-19 epidemic in Maranhão began with large cities, more integrated into economic and air transport networks. Early in the pandemic, the cities of São Luís and Imperatriz reported the highest number of cases and deaths within the state. São Luís was the first Brazilian capital to enforce lockdown and one of the first to reopen non-essential retails. Thus, flattening the curves and reopening the economy led to a decrease in the perceived risk of virus transmission. When compared to the rest of the state, São Luís presents better socioeconomic conditions and health service network, which may have sparked a feeling that the worst has passed and consequently reduced the population desire for the vaccine. In smaller cities, the epidemic may have outlined the negative perceptions about health and socioeconomic conditions and local healthcare systems, instilling hope for the covid-19 vaccine protective potential. International studies offered similar explanations, associating covid-19 vaccine rejection with pandemic dynamics and socioeconomic status^9^^,^^10^^,^^23^.

Many studies addressing vaccine hesitancy include within its drivers individual factors such as emotions, values, risk perceptions, knowledge, and beliefs, which depend on gender, age, and religion^11^. Although we found these factors to be associated with vaccine hesitancy, these findings differ from part of the available literature. In Turkey^23^, women hesitated more than men to the covid-19 vaccine, but no association was found in the United Kingdom^23^. A study conducted with the most populous countries in the world found men to be more likely to reject the vaccine^9^. Two surveys performed in North America^10^^,^^13^ found an association between women and vaccine hesitancy. Regardless of this heterogeneity, women may hesitate more because, while searching for information on vaccines to make health decisions for their children, they may be exposed to anti-vaccination content online^23^. A study addressing the rejection of other vaccines reported similar explanations^26^. Yet another reason for the higher vaccine hesitancy among women is that men, having a greater participation in labor markets outside their homes, may feel at a greater risk of exposure to the virus, thus wishing for more vaccine-induced immunity.

Studies on hesitancy towards a vaccine for covid-19^9^^,^^10^^,^^24^ or other diseases^8^^,^^10^ found older adults to be less likely than younger adults to present such behavior – different from what we observed in this survey. This discrepancy may be explained by the socioeconomic differences between older adults from Maranhão and those from high-income countries, as well as by how they perceive the pandemic from the media and political discourses. In Maranhão, aging occurs in situations of poverty and greater social needs than those observed in most Brazilian states. This population low functional literacy hinders their understanding about the role and importance of health measures such as self-care and immunization^27^. Moreover, social isolation may have had opposite effects in this population – on the one hand, they were less exposed to consultations and health professionals; on the other, they were more exposed to traditional news and social media coverage about the covid-19. In that context, older adults became less likely to acquire reliable information, more suitable to their understanding on health. Brazilian troubled political environment in coping with the pandemic may also have shaken older adults’ confidence in the information provided about the covid-19 pandemic and vaccine^8^^,^^10^^,^^13^^,^^24^^,^^27^.

As observed in other countries, religion also played a role in the covid-19 vaccine rejection and low compliance with non-pharmacological interventions (NPI)^13^^,^^28^. Since the pandemic onset, a considerable amount of misinformation and conspiracy theories has been spread through social media, following the coronavirus spread itself, affecting especially certain religious groups. Religious beliefs may play either constructive or harmful roles in a pandemic scenario, impacting people's behavior. In negative terms, religious fundamentalism helps spreading misinformation and preaches religious attitudes based exclusively on faith as a guarantee of protection against covid-19 (prayers, fasts, and trust in the divine will)^13^^,^^28^. Religious leaders are pivotal in establishing social norms and stimulating individual and collective responses to NPI and vaccine acceptance^13^^,^^28^. In several countries (Brazil included), discourses of denial made by religious leaders, based on conspiracy theories and misinformation, jeopardized public health communications and harmed epidemic control^28^.

In our study, we found people without self-reported symptoms to be more prone to vaccine hesitancy than those who reported experiencing any symptoms, or even associated with covid-19 diagnosis. Studies show that people without or with few symptoms may have lower perceptions of the risk, complications, or severity of covid-19 than those who experienced typical or severe forms of the disease^13^^,^^25^. Such perception may likewise reflect the access to inaccurate information regarding the pandemic and its current dynamics. The reopening of non-essential retail outlets and the discourses of some political and religious leaders, classifying the disease as of lesser clinical relevance and devaluing its epidemiological impact, may have shaped the popular notion that covid-19 is easy to control and poses but a low risk, so that immunization would not be as necessary^13^^,^^28^.

Our study has some limitations. Being a cross-sectional survey, we faced difficulties in establishing the direction of associations. However, most variables in this study were demographic, so that we may assume that vaccine hesitancy depends more on them than the other way around. In our study, education level and income were not associated with the outcome, differing from that reported in other studies in the literature^9^^,^^13^. Data on compliance with NPI and the use of public transportation during the pandemic was self-reported, thus incurring memory bias and possibly justifying the lack of association with the studied outcome. Likewise, symptoms were investigated through recall, according to the month in which they were experienced. However, symptoms frequency was investigated based on those more associated with covid-19 diagnosis – anosmia/hyposmia or ageusia/dysgeusia^16^. Regarding vaccination intention, the questionnaire was administered when vaccination had not yet begun, so that few details on how it would be performed had been disclosed by then. The survey was conducted when most retail outlets were already reopened, and not at the peak of the pandemic, which may have influenced how individuals perceive vaccine. Despite these limitations, the results arise from a representative sample of the state of Maranhão, considering its municipalities different population sizes.

### Final Remarks

Our results indicate a relevant prevalence of vaccine hesitancy in Maranhão, which is associated with individual, contextual, and clinical factors. We also identified the groups and contexts of greatest resistance, requiring special attention from public strategies to ensure wide vaccination. For that, authorities must prepare the population with more effective messages, aligning the political, religious, and health discourse around the benefits of immunization. Such actions may boost confidence, reduce vaccine resistance, and maximize its socioeconomic and public health benefits.
