# Supplementary material for: Prevalence and factors associated with covid-19 vaccine hesitancy in Maranhão, Brazil
Source: Rev Saude Publica. 2021 Apr 15;55:12. doi: 10.11606/s1518-8787.2021055003417 (PMC8046425; doi:10.11606/s1518-8787.2021055003417)
Supplement: Translation

# Translation: Prevalência e fatores associados à hesitação vacinal contra a covid-19 no Maranhão, Brasil

**Authors:** Bruno Luciano Carneiro Alves de Oliveira, Marcos Adriano Garcia Campos, Rejane Christine de Sousa Queiroz, Maria Teresa Seabra Soares de Britto e Alves, Bruno Feres de Souza, Alcione Miranda dos Santos, Antônio Augusto Moura da Silva

**Affiliations:** I São LuísMA Brasil Universidade Federal do Maranhão. Departamento de Medicina I. São Luís, MA, Brasil; II São LuísMA Brasil Secretaria Municipal de Saúde. São Luís, MA, Brasil; III São LuísMA Brasil Universidade Federal do Maranhão. Departamento de Saúde Pública. São Luís, MA, Brasil; IV São LuísMA Brasil Universidade Federal do Maranhão. Departamento de Engenharia da Computação. São Luís, MA, Brasil

**Keywords:** Infecções por Coronavirus, prevenção & controle, Recusa de Vacinação psicologia, Conhecimentos, Atitudes e Prática em Saúde, Inquéritos Epidemiológicos

## Abstract

**OBJETIVOS:**

Estimar a prevalência e fatores associados à hesitação ao uso da vacina contra o vírus SARS-CoV-2 no Maranhão, Brasil.

**MÉTODOS:**

Estudo transversal de base populacional realizado de 19 a 30 de outubro de 2020. As estimativas consideraram agrupamento, estratificação e não resposta. A seleção da amostra foi realizada em três estágios (estrato, setores censitários e domicílio). Após análise sistemática, em cada estrato foram selecionados trinta setores, totalizando 150 setores, sendo o número de domicílios em cada setor fixado em 34, totalizando 5.100 domicílios e um indivíduo por domicílio (residente pelo menos há seis meses e com um ano de idade ou mais) selecionado por amostra aleatória simples. A intenção de ser vacinado foi questionada aos participantes. Foi realizada análise descritiva (frequências ponderadas) e teste do qui-quadrado de Pearson para verificar associação univariada entre as variáveis independentes e o desfecho (p < 0,05). Realizou-se análise multivariada robusta utilizando-se modelagem hierarquizada em três níveis.

**RESULTADOS:**

Foram entrevistados 4.630 indivíduos. A prevalência de hesitação vacinal foi de 17,5% (IC95% 16,1–19,1%). Após ajuste final do modelo, a hesitação vacinal foi estatisticamente maior entre moradores das cidades de Imperatriz (24,0%; RP = 1,48; IC95% 1,09–2,02) e de munícipios da Grande Ilha de São Luís (20,7%; RP = 1,34; IC95% 1,02–1,76), pessoas do sexo feminino (19,8%; RP = 1,44; IC95% 1,20–1,75), idosos (22,8%; RP = 1,79; IC95% 1,30–2,46), pertencentes às religiões de denominação evangélica (24,1%; RP = 1,49; IC95% 1,24–1,79) e entre aqueles sem relato de sintomas (18,6%; RP = 1,24; IC95% 1,02–1,51). Outras características socioeconômicas e demográficas, assim como variáveis relacionadas ao mercado de trabalho, comportamentos e condições de saúde dos entrevistados, não tiveram diferença estatística.

**CONCLUSÃO:**

A prevalência de hesitação vacinal no Maranhão e sua associação com fatores individuais, contextuais e clínicos revelam os grupos e contextos mais resistentes e que devem merecer atenção especial das estratégias públicas para garantir a ampla vacinação.

## INTRODUÇÃO

No Brasil, ao longo da pandemia, ações estratégicas foram mobilizadas por governos estaduais e municipais para garantir o controle da exposição ao vírus SARS-CoV-2. Uma extensa lista de medidas não farmacológicas (MNF) foi implementada para minimizar a magnitude da doença no país^1^. Contudo, uma solução permanente é mais provável de ser alcançada com o desenvolvimento, disponibilidade e implementação de vacinas eficazes, seguras e de alta qualidade contra o vírus^2^.

A vacinação é uma das formas mais econômicas de se evitar doenças. Atualmente, estima-se que evite 2 a 3 milhões de mortes por ano e que mais 1,5 milhão poderia ser evitado se a cobertura global para vacinas disponíveis melhorasse^3^. O Brasil tem um dos maiores programas públicos de imunização do mundo, com oferta, em calendário básico de rotina e campanhas, de uma extensa lista de vacinas e outros imunobiológicos especiais para públicos específicos. Porém, a alta cobertura, que era uma das suas principais características, caiu nos últimos anos. Em 2019, foram aplicadas 89.776.476 milhões de doses de vacinas. As coberturas variaram de 61,2% no estado do Rio de Janeiro e 63,2% no Maranhão a 90,0% no Mato Grosso do Sul. Em 2020, fatores relacionados à pandemia causaram suspensão da vacinação, reduzindo o número de doses (n = 57.519.127) e taxas de vacinação (32,2% no Amapá, 39,2% no Maranhão e 66,1% no Distrito Federal)^4^, gerando novos surtos de doenças evitáveis por uso de imunobiológicos^5^.

Os benefícios epidemiológicos e socioeconômicos dos programas de imunização são bem conhecidos. Mas o debate sobre a hesitação vacinal (a relutância, indecisão ou recusa em se vacinar, apesar da disponibilidade de vacinas nos serviços de saúde) tem crescido em todo o mundo, tornando-se questão central para os programas de imunização devido à ameaça global que representa a reversão do progresso feito no combate às doenças imunopreveníveis^6^^-^^8^. As razões associadas à hesitação vacinal são complexas e podem variar em forma e intensidade com base em quando e onde ocorre, qual vacina está envolvida e que público deveria usá-las^9^^,^^10^. Assim, desde 2019, a Organização Mundial da Saúde incluiu a hesitação vacinal como uma entre dez ameaças à saúde global e, junto com outras instituições não governamentais, vem recomendando estratégias para sua mitigação^3^^,^^6^.

Contra o novo coronavírus, há um relevante número de vacinas em diferentes fases de avaliação. Algumas estão em estágio mais avançado de testagem, permitindo seu uso populacional entre o final de 2020 e os primeiros meses de 2021 na maioria dos países de renda média e alta^11^^,^^12^. Apesar do avanço, na pandemia da covid-19, polarizações políticas, teorias conspiratórias, movimento antivacina e preocupações relacionadas às vacinas contra covid-19 aumentaram rapidamente nas redes sociais e nas mídias tradicionais. Informações não verificadas e incompletas, rumores e “memes” sobre essas vacinas e a origem do coronavírus estão alcançando muitas pessoas mais rápido do que informações científicas complexas^11^^,^^13^^,^^14^ e podem afetar a confiança e aceitação desses imunizantes^9^^,^^11^. Embora novas vacinas possam ter aumentado as esperanças de retornar à normalidade pré-covid-19, elas também levantam questões sobre efeitos desconhecidos e especulação sobre a potencial vacinação compulsória^11^^,^^13^^,^^14^.

Logo, dentro dos países, as autoridades devem lidar com questões éticas e sanitárias complexas para resolver o problema da hesitação vacinal, dados os recursos disponíveis. Por isso, precisam conhecer quais grupos são mais resistentes ao uso da vacina e que ações estratégicas devem ser implementadas para preparar a população para a vacinação, a fim de alcançar elevadas e homogêneas coberturas entre grupos e localidades diferentes. Conhecer a prevalência e características associadas à hesitação vacinal no contexto dos estados brasileiros pode ser bastante útil para a efetividade de campanhas e programas de vacinação contra a covid-19.

Portanto, este estudo estimou a prevalência e os fatores associados à hesitação vacinal ao uso da vacina contra a covid-19 em inquérito sobre infecção pelo vírus SARS-CoV-2 realizado no estado do Maranhão, Brasil.

## MÉTODOS

### Tipo de Estudo e População

Estudo transversal com dados do inquérito sorológico domiciliar de base populacional intitulado “Prevalência de infecção pelo vírus SARS-CoV-2 no Maranhão, Brasil”, realizado em cooperação entre a Universidade Federal do Maranhão e a Secretaria de Estado da Saúde do Maranhão no período de 19 a 30 de outubro de 2020. Os municípios do Maranhão foram divididos em cinco estratos, segundo o porte populacional municipal de 2019 do IBGE^15^ Grande Ilha, menos de 20.000 habitantes, de 20.000 a 100.000 habitantes, mais de 100.000 habitantes, e a cidade de Imperatriz (segunda maior em população e economia do estado). A Grande Ilha incluiu a capital São Luís e mais três cidades vizinhas.

### Amostra

Para o cálculo do tamanho da amostra, adotou-se a prevalência de infectados por SARS-Cov-2 estimada no primeiro inquérito realizado no estado do Maranhão^16^. Em cada estrato, o cálculo do tamanho da amostral foi estimado a partir da seguinte equação:

$$n=\frac{N}{N-1}*P*Q*\frac{1}{CV^{2*}P^{2*}\frac{P^{*}Q}{N-1}},$$

Sendo *N* a população em cada estrato, *P* a prevalência e *CV* o coeficiente de variação das estimativas da prevalência esperada dentro dos estratos. Considerou-se efeito de delineamento igual a 2. A amostra do estudo foi de 5.001 indivíduos: 872 no Estrato 1 (quatro municípios), 1.236 no Estrato 2 (122 municípios), 612 no Estrato 3 (85 municípios), 1.022 no Estrato 4 (cinco municípios) e 1.021 no Estrato 5 (um município).

A seleção amostral foi realizada em três estágios. No primeiro, em cada estrato foram selecionados setores censitários. No segundo, dentro dos setores, os domicílios. No terceiro, dentro do domicílio, apenas um indivíduo residente.

Em cada estrato a seleção dos setores foi obtida a partir de amostra aleatória sistemática, proporcional ao número de domicílios particulares permanentes. Foram selecionados trinta setores, totalizando 150 setores. Setores com menos de 200 domicílios no censo de 2010 foram agrupados com outros, respeitando a continuidade desses setores, de forma que cada setor agrupado tivesse pelo menos 200 domicílios. O número de setores e de domicílios foi obtido do Censo Demográfico 2010^17^.

A seleção dos domicílios em cada um dos 150 setores ou agrupamentos foi obtida por amostra sistemática. O número de domicílios em cada setor ou agrupamento foi fixado em 34, totalizando 5.100 unidades domiciliares. Dentro de cada domicílio, um indivíduo foi selecionado a partir de amostra aleatória simples, totalizando 5.100 indivíduos. Essa seleção foi feita a partir de uma lista de moradores elegíveis construída no momento da entrevista (residente pelo menos há seis meses no domicílio e comum ano de idade ou mais). A amostra final do inquérito alcançou taxa de resposta de 65,4% (n = 4.630 indivíduos). O peso amostral final considerou os três estágios de seleção e a taxa de resposta.

### Coleta dos Dados

Os dados foram coletados por meio da aplicação de questionário e coleta de 5mL de sangue do indivíduo sorteado. O registro dos dados foi realizado em aplicativo a partir de dispositivo móvel, utilizando-se a plataforma *EpiCollect*^18^.

As equipes de coleta de dados eram compostas por um técnico de laboratório e um entrevistador, que utilizavam equipamentos de proteção individual. A coleta de sangue foi obtida para detectar a presença dos anticorpos totais (IgM, IgG e IgA) contra o SARS-CoV-2 por meio de teste sorológico a partir do método de imunoensaio por eletroquimioluminescência (Eclia), com o auxílio do analisador de imunoensaio cobas^®^ módulo e 601 (Roche Diagnostics) e reagentes Elecsys^®^ Anti-SARS-CoV-2 (Roche Diagnostics)^19^^,^^20^.

### Variáveis em Estudo

Neste estudo, a variável desfecho foi a hesitação vacinal ao uso da vacina contra o SARS-Cov-2, avaliada pela pergunta: “Caso estivesse disponível para a população, tomaria a vacina contra a covid-19?”. As respostas possíveis eram “sim”, “não” e “não sabe”. No caso das crianças, considerou-se a decisão do responsável. As categorias de resposta “não” e “não sabe” foram agregadas como “hesitação vacinal”, por refletirem recusa ou indecisão sobre o uso.

Utilizou-se um conjunto de variáveis independentes. As variáveis socioeconômicas e demográficas foram: sexo, faixa etária em anos (1–19, 20–59, ≥ 60), cor/raça (branca, parda, preta), escolaridade (até ensino fundamental II completo, médio completo, superior completo), renda familiar em reais (< 1.000, de 1.000 a < 2.000, ≥ 2.000), posse de plano de saúde (sim, não), religião (católica, evangélica, não tem, outras), número de moradores no domicílio (1, 2, ≥ 3) e uso de transporte coletivo durante a pandemia (não, sim). As relacionadas ao mercado de trabalho após o surgimento da pandemia foram: continua com trabalho presencial (sim, não, não trabalhava fora de casa), trabalho remoto mesmo que parcial (sim, não, não trabalhava), perdeu emprego ou teve redução de rendimentos (sim, não, não trabalhava), recebeu Bolsa Família (sim, não), recebeu auxílio emergencial (sim, não) e recebeu seguro-desemprego (sim, não, não trabalhava). Já as variáveis de comportamentos e condições de saúde foram: ter se mantido em adesão às medidas não farmacológicas desde o início da pandemia (utilizando março de 2020 como referência) até a data do inquérito (outubro de 2020), ou seja, isolamento social (sim, não), uso de máscaras (sim, não), lavagem das mãos (sim, não) e distanciamento físico (sim, não); frequência de sintomas^6^ possivelmente relacionados à covid-19 (sem sintomas, de 1 a 2, ≥ 3); realização de exames diagnósticos para SARS-CoV-2 (RT-PCR, teste rápido ou sorologia) antes do inquérito (sim, não); e sorologia para o vírus SARS-CoV-2 identificada no inquérito (positiva, negativa).

### Análise Estatística

A análise estatística foi realizada no *software* Stata^®^ versão 14, considerando-se as características do plano amostral complexo e a ponderação da amostra. Estimou-se a prevalência da hesitação vacinal e respectivos intervalos de confiança a 95% (IC95%) segundo as variáveis independentes, com teste do qui-quadrado de Pearson para verificar a associação univariada entre elas, ao nível de significância de 5%. Realizou-se análise multivariada de Poisson, utilizando-se modelagem hierarquizada (Figura). As variáveis explicativas foram dispostas em três níveis: distal (características socioeconômicas e demográficas), intermediário (características relacionadas ao trabalho após o surgimento da pandemia), e proximal (comportamentos e condições de saúde). As variáveis foram analisadas em bloco, e as que apresentaram p < 0,05 foram mantidas nas análises com o acréscimo das variáveis do bloco seguinte até o modelo final, sendo apresentadas aquelas com p < 0,05. Foi estimada a razão de prevalência (RP) e o IC95% por meio de regressão de Poisson com variância robusta (α = 5%). Foram apresentadas as estimativas para as variáveis em cada bloco.

**Figura:** Modelo hierarquizado para análise dos fatores associados à hesitação vacinal ao uso da vacina contra o novo coronavírus, caso ela já estivesse disponível, segundo o inquérito sobre infecção pelo vírus SARS-CoV-2 no Maranhão, Brasil, 2020.

### Aspectos Éticos

O referido inquérito foi aprovado pelo Comitê de Ética em Pesquisa do Hospital Carlos Macieira da Secretaria de Estado da Saúde do Maranhão sob o número CAAE 34708620.2.0000.8907.

## RESULTADOS

A prevalência de hesitação ao uso da vacina contra o vírus SARS-CoV-2 foi de 17,5% (IC95% 16,1–19,1). Essa prevalência foi estatisticamente maior (p = 0,0036) no município de Imperatriz (24,0%; IC95% 18,1–31,1) e naqueles que formam a Grande Ilha de São Luís (20,7%; IC95% 17,6–24,3) do que entre as cidades com menos de 20.000 habitantes (14,0%; IC95% 11,7–16,6). A hesitação vacinal também foi estatisticamente maior entre pessoas do sexo feminino (19,8%; IC95% 17,9–21,9; p = 0,0001), idosos (22,8%; IC95% 19,5–26,4; p = 0,0003) e seguidores de religião evangélica (24,1%; IC95% 20,7–27,9; p = 0,0001) em relação aos seus grupos de comparação. Para as demais características socioeconômicas e demográficas, a prevalência de hesitação vacinal seguiu valores próximos à estimativa estadual, porém sem associação estatística (p > 0,05), conforme apresentado na Tabela 1.

**Tabela 1:** Prevalência da hesitação vacinal ao uso da vacina contra o novo coronavírus, caso ela estivesse já disponível para a população, segundo características socioeconômicas e demográficas dos entrevistados no inquérito sorológico no Maranhão, Brasil, 2020.

Variáveis		Hesitação vacinal				p^a^
		Sim		Não		
		%	IC95%	%	IC95%	
População geral		17,5	16,1–19,1	82,5	80,9–83,9	
Estrato das cidades						
	< 20 mil habitantes	14,0	11,7–16,6	86,0	83,4–88,3	0,0036
	20 a 100 mil habitantes	16,6	14,1–19,5	83,4	80,5–85,9	
	> 100 mil habitantes	16,9	14,4–19,7	83,1	80,3–85,6	
	Imperatriz	24,0	18,1–31,1	76,0	68,9–81,9	
	Grande Ilha^b^	20,7	17,6–24,3	79,3	75,7–82,4	
Sexo						
	Feminino	19,8	17,9–21,9	80,2	78,1–82,2	0,0001
	Masculino	13,2	11,3–15,5	86,8	84,5–88,7	
Faixa etária (em anos)						
	0–19	13,1	10,0–16,9	87,9	83,1–90,0	0,0003
	20–59	16,7	14,9–18,7	83,3	81,3–85,1	
	≥ 60	22,8	19,5–26,4	77,2	73,6–80,5	
Cor/raça^c^						
	Branca	17,4	14,2–21,2	82,6	78,8–85,8	0,166
	Parda	18,0	16,1–20,0	82,0	80,0–83,9	
	Preta	14,8	11,8–18,5	85,2	81,5–88,2	
Escolaridade^d^						
	Até ensino fundamental II completo	17,8	16,0–19,8	82,2	80,2–84,0	0,231
	Ensino médio completo	18,2	15,5–21,4	81,8	78,6–84,6	
	Ensino superior completo	13,7	10,3–18,0	86,3	82,0–89,7	
Renda familiar (R$)^d^						
	< 1.000	15,7	13,0–19,0	84,3	81,0–87,0	0,3731
	De 1.000 a < 2.000	18,6	16,4–21,0	81,4	79,0–83,6	
	≥ 2.000	17,5	14,9–20,3	82,5	79,7–85,1	
Possui plano de saúde						
	Não	17,4	15,9–19,0	82,6	81,0–84,1	0,4879
	Sim	19,5	14,2–26,1	80,5	73,9–85,8	
Religião						
	Católica	15,2	13,6–17,0	84,8	83,0–86,4	0,0001
	Evangélica	24,1	20,7–27,9	75,9	72,1–79,3	
	Não tem	20,0	14,2–27,4	80,0	72,6–85,8	
	Outras	12,7	5,6–26,3	87,3	73,7–94,4	
Número de moradores no domicílio						
	1	21,0	17,2–25,3	79,0	74,7–82,8	0,120
	2	19,1	16,5–21,9	80,9	78,1–83,5	
	≥ 3	16,8	15,0–18,8	83,2	81,2–85,0	
Usou transporte coletivo durante a pandemia						
	Não	16,4	14,5–18,4	83,6	81,6–85,5	0,074
	Sim	19,2	16,9–21,6	80,8	78,4–83,1	

IC95%: intervalo de confiança de 95%.

a Teste do qui-quadrado de Pearson.

b Inclui a capital, São Luís.

c Excluídas raças amarela e indígena por baixa frequência.

d n diferente de 4.563.

Não se observou associação estatisticamente significante (p *>* 0,05) da prevalência de recusa vacinal com as características relacionadas ao mercado de trabalho e a vulnerabilidade socioeconômica dos entrevistados com o surgimento da pandemia (Tabela 2). De forma similar, a Tabela 3 apresenta os comportamentos e condições de saúde dos entrevistados em relação à hesitação vacinal, sem associação estatística com nenhuma das variáveis testadas. A prevalência da recusa para essas variáveis foi próxima à estimada para todo o estado.

**Tabela 2:** Prevalência da hesitação vacinal ao uso da vacina contra o novo coronavírus, caso ela estivesse já disponível para a população, segundo características relacionadas ao mercado de trabalho após o surgimento da pandemia dos entrevistados no inquérito sorológico no Maranhão, Brasil, 2020.

Variáveis		Hesitação vacinal				p^a^
		Sim		Não		
		%	IC95%	%	IC95%	
Continua com trabalho presencial						
	Não	17,5	15,1–20,2	82,5	79,8–84,9	0,0564
	Sim	13,7	10,8–17,2	86,3	82,8–89,2	
	Não trabalhava fora de casa	18,8	16,7–21,2	81,2	78,8–83,3	
Trabalho remoto, mesmo que parcial						
	Não	17,1	14,9–19,7	82,9	80,3–85,1	0,4353
	Sim	15,1	11,4–19,7	85,0	80,3–88,7	
	Não trabalhava	18,2	16,2–20,5	81,8	79,5–83,4	
Perdeu emprego ou teve redução de rendimentos						
	Não	17,2	15,1–19,6	82,8	80,4–84,9	0,8049
	Sim	16,5	12,5–21,4	83,5	78,7–87,5	
	Não trabalhava	18,0	15,8–20,4	82,0	79,6–84,2	
Recebeu Bolsa Família						
	Não	17,9	16,1–19,8	82,1	80,2–83,9	0,5468
	Sim	16,9	14,4–19,7	83,1	80,3–85,6	
Recebeu auxílio emergencial						
	Não	18,5	16,3–21,0	81,5	79,1–83,7	0,2632
	Sim	16,8	14,9–18,9	83,2	81,1–85,1	
Recebeu seguro-desemprego						
	Não	16,3	14,5–18,3	83,7	81,7–85,5	0,1791
	Sim	14,9	0,7–30,4	85,1	69,6–93,5	
	Não trabalhava	19,4	17,0–22,0	80,6	78,0–83,0	

1 IC95%: intervalo de confiança de 95%.

a Teste do qui-quadrado de Pearson.

**Tabela 3:** Prevalência da hesitação vacinal ao uso da vacina contra o novo coronavírus, caso ela estivesse já disponível para a população, segundo comportamentos e condições de saúde dos entrevistados no inquérito sorológico no Maranhão, Brasil, 2020.

Variáveis		Hesitação vacinal				p^a^
		Sim		Não		
		%	IC95%	%	IC95%	
Manteve-se em adesão às medidas não farmacológicas desde o início da pandemia até a data do inquérito						
Isolamento social^b^						
	Não	18,1	16,3–20,0	81,9	80,0–83,7	0,2968
	Sim	16,3	13,8–19,1	83,7	80,9–86,2	
Uso de máscaras^c^						
	Não	18,1	16,2–20,2	81,9	79,8–83,8	0,3828
	Sim	16,7	14,6–19,3	83,3	80,9–85,4	
Lavagem das mãos^d^						
	Não	17,9	16,1–20,0	82,1	80,0–84,0	0,4760
	Sim	16,8	14,6–19,3	83,2	80,7–85,4	
Distanciamento físico^e^						
	Não	17,4	15,7–19,2	82,6	80,8–84,3	0,7608
	Sim	17,9	15,2–21,0	82,1	79,4–84,8	
Recebeu diagnóstico médico^f^						
	Não	17,4	15,9–19,0	82,6	81,0–84,1	0,5401
	Sim	19,3	13,8–26,4	80,7	73,6–86,2	
Frequência de sintomas possivelmente relacionados à covid-19^g^						
	Sem sintomas	18,6	16,6–20,9	81,4	79,1–83,4	0,3990
	Um a dois sintomas	16,4	12,5–21,2	83,6	78,8–87,5	
	Três ou mais sintomas	16,6	14,3–19,1	83,4	80,9–85,7	
Já realizou algum exame diagnóstico para SARS-CoV-2^h^ antes do inquérito						
	Não	17,7	16,1–19,3	82,3	80,7–83,9	0,6455
	Sim	16,5	12,4–21,6	83,5	78,4–87,6	
Sorologia para o vírus SARS-CoV-2 identificada no inquérito						
	Positiva	18,8	16,4–21,5	81,2	78,5–83,6	0,1894
	Negativa	16,7	15,0–18,7	83,3	81,3–85,1	

IC95%: intervalo de confiança de 95%.

a Teste do qui-quadrado de Pearson.

b Nunca sai de casa ou quase nunca sai – no máximo uma saída a cada 15 dias.

c Usa em todas as saídas e não retira ou quase não retira do rosto.

d Higieniza seis vezes ou mais por turno.

e Não se aproxima nunca ou quase nunca de outras pessoas a menos de 1,5 metros.

f De suspeita de covid-19.

g Foram considerados os seguintes sintomas: febre, calafrios, dor de garganta, tosse, dispneia, anosmia, ageusia, diarreia, náuseas/vômitos, dor de cabeça, fadiga e mialgia. Eles foram classificados entre: sem sintomas; presença de um a dois sintomas, desde que estes não fossem anosmia/hiposmia ou ageusia/disgeusia; três ou mais sintomas (tendo entre eles a presença de anosmia/hiposmia ou ageusia/disgeusia).

h RT-PCR, teste rápido ou sorologia.

Após ajuste no modelo multivariado hierarquizado, observou-se que a chance de hesitação vacinal foi estatisticamente maior entre moradores das cidades de Imperatriz (RP = 1,48; IC95% 1,09–2,02) e Grande Ilha (RP = 1,34; IC95% 1,02–1,76), pessoas do sexo feminino (RP = 1,44; IC95% 1,20–1,75), idosos (RP = 1,79; IC95% 1,30–2,46), pertencentes às religiões evangélicas (RP = 1,49; IC95% 1,24–1,79) e entre os sem sintomas durante a pandemia (RP = 1,24; IC95% 1,02–1,51) (Tabela 4).

**Tabela 4:** Fatores associados à hesitação vacinal ao uso da vacina contra o novo coronavírus, caso ela estivesse já disponível para a população, entre entrevistados na Fase II do inquérito sorológico no Maranhão, Brasil, 2020.

Variáveis		Bloco distal		Bloco distal + intermediário		Bloco distal + intermediário + proximal	
		RP^a^	IC95%	RP^b^	IC95%	RP^c^	IC95%
Estrato das cidades							
	< 20 mil habitantes	1,00	-----				
	20 a 100 mil habitantes	1,11	0,88–1,40				
	> 100 mil habitantes	1,15	0,91–1,46				
	Imperatriz	1,48	1,09–2,02				
	Grande Ilha	1,34	1,02–1,76				
Sexo							
	Masculino	1,00	-----				
	Feminino	1,44	1,20–1,75				
Faixa etária (em anos)							
	0–19	1,00	-----				
	20–59	1,29	0,95–1,74				
	≥ 60	1,79	1,30–2,46				
Religião							
	Católica	1,00	-----				
	Evangélica	1,49	1,24–1,79				
	Não tem	1,38	0,96–2,00				
	Outras	0,85	0,40–1,82				
Frequência de sintomas possivelmente relacionados à covid-19^d^							
	Três ou mais sintomas					1,00	----
	Um a dois sintomas					1,08	0,80–1,47
	Sem sintomas					1,24	1,02–1,51

1 IC95%: intervalo de confiança; RP: razão de prevalência obtida por regressão de Poisson.

a Ajustadas uma pelas outras.

b Ajustados para sexo, estrato, faixa etária, raça, religião, número de moradores e uso de transporte coletivo. Não foi observada associação com nenhuma das variáveis do bloco intermediário.

c Ajustada por sexo, estrato, faixa etária, religião, uso das medidas não farmacológicas, testagem para covid-19 antes e pelo inquérito.

d Foram considerados os seguintes sintomas: febre, calafrios, dor de garganta, tosse, dispneia, anosmia, ageusia, diarreia, náuseas/vômitos, dor de cabeça, fadiga e mialgia. Eles foram classificados entre: sem sintomas; presença de um a dois sintomas, desde que estes não fossem anosmia/hiposmia ou ageusia/disgeusia; três ou mais sintomas (sendo entre eles a presença de anosmia/hiposmia ou ageusia/disgeusia).

## DISCUSSÃO

Os resultados indicaram que a maioria dos entrevistados referiu desejo de tomar a vacina contra o vírus SARS-CoV-2, caso ela estivesse disponível. Porém também foi observada relevante prevalência de hesitação vacinal. No modelo de ajuste, essa prevalência esteve associada a características individuais, contextuais e clínicas, tais como ser do sexo feminino, ser idoso, seguir uma religião evangélica, residir nos dois estratos de cidade do estado com maior porte populacional e não apresentar sintomas mais relacionados à covid-19 durante a pandemia.

A tomada de decisão em torno da vacinação é um fenômeno comportamental complexo em relação aos seus determinantes. Envolve aspectos culturais, geográficos, psicossociais, econômicos, religiosos, políticos, fatores cognitivos e de gênero. As razões para a hesitação vacinal se encaixam em três categorias inter-relacionadas: falta de confiança (na eficácia, na segurança, no sistema de saúde que fornece as vacinas ou nas motivações dos gestores e formuladores de políticas para recomendá-las), complacência (baixa percepção do risco de adquirir doenças imunopreveníveis, de forma que a vacinação não seria necessária) e falta de conveniência (considera a disponibilidade, acessibilidade e o apelo dos serviços de imunização, incluindo tempo, lugar, idioma e contextos culturais)^21^^,^^22^.

Em outros países, pesquisas também indicaram prevalências relevantes de hesitação vacinal contra a vacina do SARS-CoV-2. Um inquérito com amostra aleatória de 13.426 pessoas de 19 países (cuja soma das populações corresponde a 55% da população mundial) verificou prevalência de 28,5%, variando de 11,4% na China a 45,1% na Rússia^9^. O Brasil teve a segunda menor prevalência estimada nesse estudo (14,7%), abaixo também da estimada em outros países de renda média avaliados (Índia, México e África do Sul)^9^ e da encontrada no inquérito no Maranhão. Verificou-se também prevalência de 14,0% na Turquia^23^, de 31,0% no Reino Unido^23^ e de 33,0%^10^ a 42,4%^24^ nos EUA. Já em Israel^25^, estimou-se prevalência de 25,0% na população geral, 22,0% em médicos e 39,0% em enfermeiros.

Esses resultados apontam variações importantes na hesitação vacinal segundo os contextos avaliados. Nesta pesquisa, os estratos com a capital e segunda maior e mais rica cidade do estado Maranhão apresentaram as maiores prevalências e chances de recusa vacinal em relação aos estratos de menor porte populacional, com valores próximos aos observados em outros países, como EUA, Coreia do Sul, México, Índia e Espanha^9^. Um inquérito nos EUA também observou notável variação geográfica da prevalência de rejeição à vacina da covid-19 (25,0% a 50,0%) dentro do país^10^. Tais variações podem decorrer das diferenças na dinâmica da epidemia em cada estrato de cidade, situação socioeconômica e acesso à saúde de suas populações. No Maranhão, a epidemia da covid-19 começou pelas grandes cidades, que apresentam maior integração aos circuitos econômicos e de transporte aéreo. As cidades de São Luís e Imperatriz apresentaram, ao longo dos primeiros meses da pandemia, maior volume de casos e óbitos. São Luís foi a primeira capital do país a passar por *lockdown* e uma das primeiras a ter reabertura de serviços não essenciais. Assim, a percepção populacional sobre o risco da transmissão do vírus passou a diminuir com o achatamento das curvas de casos e óbitos e reabertura econômica. O contexto de melhores condições socioeconômicas e rede de serviços de saúde em relação ao restante do estado pode ter gerado sensação de que o pior da pandemia já havia passado, resultando em redução do desejo pela vacina. Por outro lado, nas menores cidades, a epidemia pode ter destacado a percepção ruim sobre as condições de saúde, socioeconômicas e dos sistemas de saúde locais, gerado maiores esperanças de proteção da vacina contra a covid-19. Explicações semelhantes foram levantadas por estudos internacionais que associaram a rejeição à vacina da covid-19 com a dinâmica da pandemia e a situação socioeconômica^9^^,^^10^^,^^23^.

Existe um grande número de estudos sobre hesitação vacinal, destacando entre seus *drivers* fatores individuais tais como emoções, valores, percepções de risco, conhecimentos e crenças. Estes fatores dependem do sexo, idade e religião^11^. Neste estudo, esses aspectos se mostraram associados à hesitação vacinal. Porém esses achados divergem de parte da literatura disponível. Em relação à vacina contra a covid-19, na Turquia^23^, mulheres hesitaram mais do que homens; já no Reino Unido, não houve associação^23^. Entre os países mais populosos do mundo, homens apresentaram maior probabilidade de rejeitar a vacina^9^. Em dois inquéritos norte-americanos^10^^,^^13^, verificou-se associação do sexo feminino com essa hesitação vacinal. Apesar dessa heterogeneidade, especula-se que uma das razões para mulheres hesitarem mais seja que, como elas são mais propensas a tomar decisões de saúde para seus filhos, também podem ser mais propensas a buscar informações sobre vacinas e ser expostas a conteúdo antivacinação on-line^23^. Explicações semelhantes já foram atribuídas à rejeição de outras vacinas^26^. Outra razão é que homens, por terem maior inserção no mercado de trabalho fora do lar, podem se sentir mais expostos aos riscos associados ao vírus, e por isso desejam mais a proteção induzida pela vacina.

Estudos disponíveis sobre hesitação vacinal contra a covid-19^9^^,^^10^^,^^24^ ou outras doenças^8^^,^^10^ mostram idosos menos propensos a esse comportamento do que adultos, o oposto do observado neste inquérito. As divergências podem decorrer das diferenças socioeconômicas entre os idosos maranhenses em relação aos de países de alta renda e a percepção dos idosos sobre a pandemia a partir das mídias e dos discursos políticos. O envelhecimento no Maranhão ocorre em situações de pobreza e de maiores necessidades sociais do que as observadas na maioria dos estados brasileiros. O baixo letramento funcional nessa população dificulta a compreensão sobre a importância e a função de medidas de saúde, como o autocuidado e a vacina^27^. Ademais, o isolamento social pode ter produzido efeitos opostos nessa população. De um lado, os tornaram menos expostos às consultas regulares e aos profissionais de saúde, e de outro, mais expostos à cobertura da covid-19 nas redes de notícias tradicionais e mídias sociais. Logo, tornaram-se menos propensos a receber informações confiáveis e mais adequadas aos seus níveis de compreensão em saúde. Somado a isso, o conturbado contexto político no Brasil no enfrentamento da pandemia pode ter reduzido a confiança dos idosos nas informações recebidas sobre a pandemia e vacina^8^^,^^10^^,^^13^^,^^24^^,^^27^.

Como observado em outros países, a religião foi também referida como razão para rejeição à vacina da covid-19 e menor adesão às MNF^13^^,^^28^. Desde o início da pandemia, um grande volume de informações equivocadas e teorias da conspiração tem sido espalhado por meio de mídias sociais em velocidade maior do que o próprio espalhamento do novo coronavírus, afetando mais certos grupos religiosos. Em pandemias, religiões podem desempenhar papéis diferentes (construtivo ou prejudicial) que impactam o comportamento das pessoas. Em termos negativos, o fundamentalismo religioso ajuda a espalhar desinformação e prega atitudes religiosas baseadas exclusivamente na fé como garantia de proteção contra o SARS-CoV-2 (orações, jejuns e confiança na vontade divina)^13^^,^^28^. Líderes religiosos têm papel-chave no estabelecimento de normas sociais e no estímulo de respostas individuais e coletivas para adesão às MNF e aceitação das vacinas^13^^,^^28^. Em vários países, entre eles o Brasil, discursos negacionistas de líderes religiosos baseados em teorias conspiratórias e más informações prejudicaram as mensagens de saúde pública e causaram danos no controle epidemiológico da pandemia^28^.

Este estudo apontou que pessoas sem autorrelato de sintomas foram mais propensas à hesitação vacinal do que as que apresentaram alguns sintomas ou sintomas mais associados ao diagnóstico da covid-19. Estudos mostram que pessoas sem sintomas ou com poucos sintomas, em relação às que apresentaram formas mais típicas ou graves da doença, podem ter menor percepção dos riscos, complicações ou da severidade da covid-19^13^^,^^25^. Essa percepção pode ser também reflexo do acesso às informações incorretas em relação à pandemia e sua atual dinâmica. A reabertura de serviços não essenciais e discurso de alguns líderes políticos e religiosos desvalorizando o impacto epidemiológico da doença e classificando-a como doença de menor importância clínica podem ter moldado as percepções populares de que a doença é de fácil controle e baixo risco e que, portanto, vacinas não seriam tão necessárias^13^^,^^28^.

Esses resultados apresentam limitações. Estudos transversais podem ter dificuldades de estabelecer a direção das associações. Contudo, as variáveis associadas neste estudo foram, em maior parte, demográficas. Torna-se consistente supor que a hesitação vacinal depende mais delas do que o contrário. Escolaridade e renda não se mostraram associadas ao desfecho. Esse achado diverge de parte da literatura disponível^9^^,^^13^. A forma como foram colhidas as informações sobre a adesão às MNF e o uso de transportes coletivos ao longo da pandemia incorre em viés de memória, o que pode explicar a ausência de associação com o desfecho em estudo. Os sintomas também foram investigados por meio da recordação, segundo o mês em que estiveram presentes. Porém, para averiguar a frequência dos sintomas, foram usados os mais associados ao diagnóstico da covid-19, anosmia/hiposmia ou ageusia/disgeusia^16^. Sobre a intenção de tomar a vacina, a pergunta foi feita quando a vacinação ainda não estava ocorrendo, com poucos detalhes divulgados sobre como seria executada. O inquérito ocorreu já com a reabertura quase completa da economia, e não no pico da pandemia. É possível que essas condições tenham mudado a percepção sobre a vacina. Apesar dessas limitações, os resultados provêm de amostra representativa de todo o Maranhão, considerando a diversidade dos portes populacionais de suas cidades.

### Considerações Finais

Os resultados apontaram relevante prevalência de hesitação vacinal no Maranhão e sua associação com fatores individuais, contextuais e clínicos. Revelaram os grupos e contextos mais resistentes e que devem merecer atenção especial das estratégias públicas para garantir a ampla vacinação. Será necessário preparar a população com mensagens mais efetivas sobre a vacina, alinhando o discurso político, religioso e de saúde em torno das vantagens associadas a ela. Essas ações podem aumentar a confiança, reduzir as resistências à vacina, e maximizar os seus benefícios socioeconômicos e à saúde coletiva.
